# Repair of Injuries to the Skull by Perforated Plates

**Published:** 1917-11

**Authors:** 


***Repair of Injuries to the Skull by Perforated Plates.*** By A. E. MITCHELL, Belfast. Abstracted from the *British Journal of Surgery*, July, 1917.]

The author speaks favorably of silver plate coverings for gaps in the skull. The plate, somewhat thinner than an ordinary visiting card, is punched with holes one eighth of an inch in diameter as close together as possible. The perforations permit the escape of blood or other fluids and so avoid compression by accumulation between the plate and the dura or brain. They aid also in fixing the plate :

Technique of the Operation :

" 1. A large flap of scalp is turned down.

" 2. The opening in the skull is explored, and foreign body is removed, adhesions are freed, and bleeding is arrested."

" 3. The periosteum is now carefully raised from the skull for about a half an inch all around the gap.

" 4. The plate, cut to the necessary size and shape (half an inch in diameter larger than the opening), is now slipped under the reflected periosteum and fixed in position by a series of catgut sutures, which, by the aid of a fully curved needle, are carried through the periosteum and out through any of the perforations in the plate, which are most convenient. In this way the plate is securely fixed in position and cannot slip. The scalp flap is now sutured in position, and a drainage tube inserted at the most dependent angle for twenty-four hours—otherwise an hematoma is likely to form. Needless to say the most rigid asepsis must be observed."

M. has found this method especially effective in reducing if not entirely stopping epileptic fits. He states that he has operated upon six cases, that in every case primary union was obtained, and that marked relief followed the operation. None of the plates has given rise to any trouble.

